# Acta Neuropsychiatrica embraces full open access: towards a new era of global knowledge sharing

**DOI:** 10.1017/neu.2024.66

**Published:** 2025-01-21

**Authors:** Livea Dornela Godoy, Gregers Wegener

**Affiliations:** 1 Pharmacology Department, Faculdade de Medicina de Ribeirão Preto, Universidade de São Paulo, Ribeirão Preto, São Paulo, Brazil; 2 Translational Neuropsychiatry Unit, Department of Clinical Medicine, Aarhus University, Aarhus, Denmark; 3 Department of Affective Disorders, Aarhus University Hospital - Psychiatry, Aarhus, Denmark

**Keywords:** Open access, neuropsychiatry, access to information, Latin America, Africa

We are excited to announce that, effective September 23rd 2024, *Acta Neuropsychiatrica* has transitioned to a fully Open Access (OA) Gold model. All articles accepted for publication will be freely available under the Creative Commons license, significantly widening the accessibility of neuropsychiatric research. This pivotal change aligns with global trends toward open science, ensuring that cutting-edge research in neuropsychiatry is available without financial or institutional barriers.

## Plan S: a vision for open science

The journal’s new fully OA model aligns with the mandates of *Plan S*, a global initiative launched by cOAlition S, which stipulates that all publicly funded research must be published in open-access journals, platforms, or repositories (CoalitionS, 2018). *Plan S* is driven by the core belief that publicly funded research should be immediately accessible, fostering innovation, transparency, and societal benefits.

*Acta Neuropsychiatrica* supports this vision by ensuring immediate, unrestricted access to all published work. Huang and colleagues highlight in *eLife* how policy mandates—such as *Plan S*—are pivotal in driving open-access adoption. An analysis of over 1,200 institutions worldwide found that institutions under strong OA mandates, such as those in the UK, achieved up to 90% OA publishing (Huang *et al*., 2020). By contrast, institutions without such policies have lagged, emphasising the importance of enforced and monitored OA initiatives to ensure broad and equitable access.

## The societal benefits of open access

By embracing a fully OA model, *Acta Neuropsychiatrica* contributes to a more inclusive global research ecosystem. Effective communication and scientific advances rely on timely exchanges. An OA international programme has the potential to democratise science by providing access to research that would otherwise be locked behind paywalls, particularly in low-GDP countries where access to journals is limited (Huang *et al*., 2020) and with targeted initiatives. Fair OA policies can reach and benefit regions where the cost of journal subscriptions remains a significant barrier to research.

Latin America has long embraced and pioneered systems for OA through the establishment of the International Cooperation Program for Open Access Scientific Communication (SciELO), which recognises and promotes journals published by universities and scientific societies (primarily operated in non-profit contexts) with certified scientific rigour and quality aligned with international standards of scientific communication (SciELO, 1997). Together with Electronic Information for Libraries, a not-for-profit organisation that works with libraries to enable access to knowledge in developing and transition economy countries in Africa, Asia Pacific, Europe, and Latin America, a group of initiatives may work to promote real transformative and ensure the sustainable development of open-access research. Initiatives such as those mentioned and AmeliCA (AmeliCA, 2018) put effort into creating a funding system that expands into creating forums, policies, interoperable repositories, educational training, new metrics, and integrative platforms. These initiatiatives show that the Global South can embrace and drive changes in the publishing system, achieving principles of diversity, equity, inclusion, and accessibility (Kuchma and Ševkušić, 2024).

*Acta Neuropsychiatrica* is committed to those principles and strives to include in our Editorial Board researchers from across the Globe through an Editorial training programme for young scholars. We are proud of such a truly neuropsychopharmacology international community, integrated from authors to reviewers and editors, which we want to expand further. By becoming fully OA, *Acta Neuropsychiatrica* opens its platform to more projects that *facilitate the sharing, managing, and utilising of knowledge and infrastructure,* enabling greater access to scholarly resources and opportunities for research advancement.

## Breaking down financial barriers for authors

A critical issue is the financial barrier posed by article processing charges (APCs), which may be prohibitive for researchers in low-resource settings. To address this, *Acta Neuropsychiatrica* and *Cambridge University Press* has implemented several mechanisms to ensure that APCs do not hinder the publication of valuable research. Through institutional agreements, grant funding, and waivers for authors without access to financial support, we ensured that every researcher, regardless of financial background, could publish in our journal. A lack of financial support has been shown to limit OA publishing, particularly in regions with limited research funding (Huang *et al*., 2020). cOAlition S acknowledges the growing need for alternative not-for-profit publishing models and has initiated initiatives for Diamond OA (research free to read *and* free to publish). *Acta Neuropsychiatrica* and *Cambridge University Press* are committed to breaking the financial barriers and promoting a genuinely equitable publishing model by offering various APC solutions. *Acta Neuropsychiatrica* and *Cambridge University Press* are also partners in *Research4Life*, a significant initiative to support institutions in low- and middle-income countries (Research4Life, 2024). Researchers affiliated with non-profit institutions in eligible nations, categorised into Groups A and B, can apply for full waivers or discounts on article processing charges (APCs). The initiative primarily includes countries in sub-Saharan Africa, where most institutions qualify for free access to a wide range of academic journals.

## Supporting global collaboration and equity

For researchers, particularly those from underrepresented regions, publishing in an OA journal such as *Acta Neuropsychiatrica* can amplify their work on a global stage. We are committed to maintaining our journal as a platform that amplifies the voices of researchers who have published their significant contributions. This platform is particularly crucial in psychiatry, as the Global South faces some of the highest prevalence rates and global burden (Huang *et al*., 2023). By making research openly available, we enhance visibility and foster cross-border collaborations that are essential in addressing the global nature of mental health challenges.

The new OA model also benefits authors by providing greater visibility and higher citation rates. It is well documented that OA articles are read and cited more frequently than those published in subscription journals, reflecting the broader dissemination of freely available research (Huang *et al*., 2020). This increased exposure is significant for early career researchers and those working in less visible fields, offering them a platform to gain recognition for their work.

In line with the efforts to enhance integration, expand dissemination, and create a platform to showcase significant neuroscience discoveries, *Acta Neuropsychiatrica* has recently joined the Neuroscience Peer Review Consortium (NPRC). NPRC is a cross-publisher alliance aiming to reduce the workload on reviewers and authors enabling transfer of manuscripts and reviews between journals, facilitating the publication of neuroscience findings and integrating the peer-review system at low cost (NPRC, 2024). Manuscripts not accepted by one journal in the Consortium can be seamlessly transferred to any other Journal in NPRC.

## Strengthening the global neuropsychiatric research community

The societal impact of transitioning to a fully OA journal extends beyond that of the individual researchers. As noted in the study by Huang *et al*. (2020), OA policies foster a culture of openness and collaboration that benefits scientific communities. OA is particularly crucial in fields such as neuropsychiatry, where the rapid dissemination of research can lead to breakthroughs in understanding and treating mental health conditions. It enables the faster integration of research findings into clinical practice, enhances interdisciplinary collaboration, and facilitates the global exchange of ideas essential for addressing complex mental health challenges.

Moreover, this study found that institutional-level interventions, such as national OA policies and funder mandates, significantly influence OA uptake. For example, UK universities that operate under strict OA policies linked to the UK Research Excellence Framework have demonstrated higher rates of repository-mediated OA publishing (Huang *et al*., 2020). This finding suggests that institutional and national policies when effectively enforced, can drive the transition to open science on a larger scale. *Acta Neuropsychiatrica* aims to play a similar role by encouraging institutions and funding bodies worldwide to prioritise OA publishing. Traditionally, we strongly encourage the use of data repositories and transparent reproducible policies, for example including the deposit of the full ARRIVE description. This elevates the quality of research and can be computed into the emerging and improved landscape of metrics of research outputs.

## Looking to the future: open access as a driver of innovation

As *Acta Neuropsychiatrica* continues its journey toward full OA, we recognise that this transition is part of a broader movement toward open science. The paper by Huang and co-workers highlights the importance of continually assessing and improving OA strategies to ensure that they remain inclusive and equitable (Huang *et al*., 2020). We are committed to working closely with the authors, institutions, and funders to ensure that our OA model remains accessible, sustainable, and beneficial to all stakeholders.

It can be foreseen that OA will become the publication norm across scientific disciplines in the coming years, leading to a transparent, collaborative, and innovative research ecosystem. By making neuropsychiatric research freely available, *Acta Neuropsychiatrica* is helping drive this transformation, ensuring that new knowledge can be applied quickly and effectively to improve mental health outcomes worldwide.

We invite authors, researchers, and institutions to join us in this effort to advance OA to neuropsychiatric research. For more information on our OA policies, APC waivers, and how to submit your work, please check our informative video (Godoy, 2024) and visit our Open Access Options page (Cambridge_University_Press, 2024).
